# Trust increases euthanasia acceptance: a multilevel analysis using the European Values Study

**DOI:** 10.1186/1472-6939-15-86

**Published:** 2014-12-20

**Authors:** Vanessa Köneke

**Affiliations:** Cologne Graduate School and SOCLIFE Research Training Group, University of Cologne, Albertus-Magnus-Platz, 50931 Cologne, Germany

**Keywords:** Euthanasia, End-of-life-decisions, Public opinion, Slippery slope, Trust, Health care, European Values Study

## Abstract

**Background:**

This study tests how various kinds of trust impact attitudes toward euthanasia among the general public. The indication that trust might have an impact on euthanasia attitudes is based on the slippery slope argument, which asserts that allowing euthanasia might lead to abuses and involuntary deaths. Adopting this argument usually leads to less positive attitudes towards euthanasia. Tying in with this, it is assumed here that greater trust diminishes such slippery slope fears, and thereby increases euthanasia acceptance.

**Methods:**

The effects of various trust indicators on euthanasia acceptance were tested using multilevel analysis, and data from the European Values Study 2008 (N = 49,114, 44 countries). More precisely, the influence of people’s general levels of trust in other people, and their confidence in the health care system, were measured—both at the individual and at the country level. Confidence in the state and the press were accounted for as well, since both institutions might monitor and safeguard euthanasia practices.

**Results:**

It was shown that the level of trust in a country was strongly positively linked to euthanasia attitudes, both for general trust and for confidence in health care. In addition, within countries, people who perceived their fellow citizens as trustworthy, and who had confidence in the press, were more supportive of euthanasia than their less trusting counterparts. The pattern was, however, not true for confidence in the state and for confidence in the health care system at the individual level. Notably, all confirmative effects held, even when other variables such as religiosity, education, and values regarding autonomy were controlled for.

**Conclusions:**

Trust seems to be a noteworthy construct to explain differences in attitudes towards euthanasia, especially when drawing cross-country comparisons. Therefore, it should be added to the existing literature on correlates of euthanasia attitudes.

**Electronic supplementary material:**

The online version of this article (doi:10.1186/1472-6939-15-86) contains supplementary material, which is available to authorized users.

Legal and public discussions about the permissibility of end-of-life-decisions like euthanasia, i.e. the intentional ending of another person’s life upon his or her request, are widespread [1–5]. While many people acknowledge the right for a self-determined death in certain circumstances, as in the case of a terminal illness [6], one common topic in these discussions is the fear of potential abuses and involuntary deaths, or at least that ill and old people might feel pressured to die [7]. In trying to connect this risk theme with the scientific literature on determinants of euthanasia attitudes, this paper analyses whether people are more willing to accept the idea of euthanasia when they think their fellow citizens and important medical and legal institutions are trustworthy.

Using data from the European Values Study [8], the influences of three kinds of trust on euthanasia attitudes are examined: trust towards other people in general; trust in the health care system; and confidence in monitoring institutions such as the press and the state. Trust in other people in general covers the perception of a benevolent human nature: this is the belief that other people in the society will abide by common ethical rules [9] and will not deliberately or knowingly do other human beings avoidable harm, but, if possible, will look after their interests [10]. Trust in in the health care system refers to having confidence that ill people are provided with all the therapies they need, thereby making involuntary euthanasia due to financial reasons less likely (c.f. [11]). Finally, confidence in the press and the state, especially with regard to their abilities in terms of justice and impartiality, might be important, because both institutions should guarantee that no abuses of euthanasia take place [12, 13].

## Background

### Background: determinates of euthanasia attitudes

Debates about euthanasia are by no means a new phenomenon. Whether the intentional ending of person´s life for his or her own good is permissible or not, has been discussed by philosophers, medical practitioners, legal professionals, and belletrists for hundreds of years [14]. In contrast, widespread discussions among members of the general public are a relatively new phenomenon. These discussions began in the 1970s, and have been especially pronounced since the start of the new millennium. Paralleling this development, polling institutes, the media, and citizen movements have been documenting levels of support and rejection for euthanasia more frequently (e.g. [15]). However, not many studies go beyond assessing opinions, to explaining them.

When it comes to the question of what influences whether individuals accept or oppose the moral and legal notion of euthanasia, only a small number of factors are known. To a large extent, religiosity and sociodemographic features such as age, ethnicity, and education have been considered as key factors, and sometimes caregiving experiences and permissiveness towards other morally loaded behaviours like abortion (see [16] for a literature review). Education and general moral permissiveness have been found to increase euthanasia acceptance, while advanced age and ethnic minority status have been found to reduce it. Religiosity usually shows a pronounced negative effect on euthanasia acceptance, for both individual religiosity and for a country’s religious climate. In addition to religious commitments, denomination effects have been found—mostly in terms of Protestants and Protestant cultures being less opposed to euthanasia than Catholics and Muslims, or Catholic and Muslim cultures, respectively. The results have been mixed with regard to gender and personal experience.

While these factors have been validated several times, few attempts have been made to analyse correlates of euthanasia attitudes systematically, and to connect the potential determinants with arguments brought forward by experts who favour or oppose euthanasia. One exception is a study by Rietjens and colleagues [17], which assessed the impacts of individuals’ perceptions of a good death, thereby establishing a connection to the pro-euthanasia-argument of ‘death with dignity’. In addition, Kemmelmeier et al. [18] liaised the pro-euthanasia-argument of self-determination by showing that individualists (people who value being unique and independent) had rather positive opinions with regard to euthanasia acceptability, and those with authoritarian mindsets (people who value strict discipline and order) had more negative attitudes. Especially noteworthy, Verbakel and Jaspers [19] tried to derive potential attitude correlates from the four main pro- and anti-euthanasia arguments—the right for self-determination, death with dignity, religiosity, and slippery slope fears—with the latter describing the fear of abuse and involuntary deaths [20]. They found that people consenting to moral relativism (deeming guidelines about what is good and evil to be context-specific instead of applying to everyone and to all circumstances, which is a kind of moral autonomy [21]), and also people living in countries with a high level of moral relativism, were more supportive of euthanasia. So were people with an internal locus of control [22], which was understood by the authors as a factor counteracting personal slippery slope fears. Their approach is laudable; however, it did not consider the defensive effect trust might have on slippery slope fears.

### The slippery slope argument

The slippery slope argument is the most prominent objection to euthanasia, with the exception of the religious demur that only God should interfere in issues of life and death. Slippery slope means that allowing, accepting, or practicing a desirable or at least neutral action A, might, in the long run, logically or practically lead to one or more undesired action(s), B. As a result, to prevent B from occurring, one has to prevent A from occurring [20, 23, 24].^a^ Concerning euthanasia, the slippery slope argument asserts that accepting one kind of euthanasia, that might be considered beneficial, will lead to other kinds, that are deemed to be unacceptable, which in turn, results in the rejection of the first kind [25].

What is considered a beneficial kind of euthanasia, and what is considered an unacceptable kind, differs between persons and cultures. Hence, the conception of what constitutes a slippery slope differs as well. The form of euthanasia often considered acceptable among the general public is that for people with a terminal illness, unbearable suffering, low life expectancy, and in response to a direct request by the patient [6]. The most condemned kind, in contrast, is clearly involuntary euthanasia, which means euthanasia against the wishes of the person going to die, or without that person’s consent [7]. This would probably constitute then end point of a potential slippery slope for almost everybody (c.f. [26, 27]). Quite similar are fears regarding what one might call pushed euthanasia, or a duty to die, describing the possibility that ill or old people might be pressured to seek euthanasia, because society or their families make them perceive themselves as a burden [28]. In-between is the acceptance of euthanasia for non-terminally ill people, old people without any severe illness, and people with psychiatric illnesses [29] (c.f. [30]), and the acceptance of so-called non-voluntary euthanasia, meaning euthanasia for people who are thought to wish to die, but who cannot request it, due to dementia for example [6, 29] (c.f. [31]).

Although poorly integrated into the research of attitudes among the general public, many papers have been written about a slippery slope in the context of euthanasia—including both theoretical ones (e.g. [25, 26, 32–34]), and empirical ones, describing conditions in countries where euthanasia has been legalised (e.g. [35–37]). Notably, countries that have legalised euthanasia have established safeguards to prevent the slippery slope from occurring. For example, in Belgium, Luxemburg, and the Netherlands it is legally required that at least two physicians have to confirm that the wish to die is voluntary and well-considered, and in all three countries a review committee has to audit granted euthanasia cases post hoc [12, 38] (c.f. [39]). Establishing legal requirements of due care is not in general a necessity, but surprisingly, the prevention of euthanasia’s slippery slope has only been looked at from this perspective of formal control (e.g. [40]). In many other fields of life, however, it has been acknowledged that whenever control is considered important, trust might offer a different way of considering the situation [41, 42].

### Trust and euthanasia

Trust is an important topic in medical ethics and end-of-life care, but has been given waning credit due to the increasing focus on autonomy [43]. Some authors dealing with slippery slope arguments generally have mentioned trust, but they have done so casually, without really analysing it. For example, Enoch [44] mentioned that proponents of the slippery slope argument mistrust others to make a distinction between A and B (e.g. voluntary and involuntary euthanasia). Similarly, Volokh [24] asked whether we should ‘accept the immediate benefits of A, and trust that even after A is enacted B will be avoided’. Van der Burg [20] remarked that people with a high level of trust in existing political and legal institutions are more confident that future developments could be stopped, should they be heading in a negative direction.

Precisely, with regard to euthanasia, several Dutch authors referred to the high level of trust in physicians felt by people in the Netherlands as one factor explaining why euthanasia has been legalised there, but not in other countries (e.g. [20, 45, 46]). Similarly, regarding the legalisation of euthanasia in Belgium, some experts pointed to the importance of high levels of trust in physicians and the health care system [47, 48]. By the same token, Cohen et al. [49] reasoned that euthanasia can be discussed seriously only in situations where there are high levels of trust in the health care system, and Battin [50] also mentioned that a rejection of euthanasia might be associated with distrust of physicians. For Turkey, opposition to euthanasia has been ascribed to a lack of confidence in health care, as well as to a lack of confidence in the political and juridical system [51].

However, links between the general public’s trust and attitude toward euthanasia has not been studied systematically. There is one study dealing with the effect of perceived discrimination by the medical system on attitudes toward euthanasia [52], and two studies dealing with the effect of trust in the health care system and in physicians, on attitudes toward physician-assisted suicide and the forgoing of life-sustaining medical treatment (not active euthanasia) [53, 54]. None of them found any effects associated with trust. However, none of these studies targeted the broader general public, and instead, included only the subgroup of ethnic minorities—one was furthermore based on cancer patients only. Incorporating a more diversified sample from the general public in the United States (US), Ward [55] found that when people over 60 years of age had confidence in medicine, there was a positive effect on euthanasia attitudes, but these findings did not apply to the whole sample population. Similarly, in another study, trust in the National Health Service (NHS) did not impact United Kingdom residents’ euthanasia attitudes [56]. In a study among the Dutch [57], trust in physicians even led to decreased acceptance of assistance in dying, which the authors explained in terms of more trust meaning less perceived need to take over control for end-of-life-decisions oneself. In the same vein, another study from the Netherlands, which dealt only with people over the age of 64, found the elderly were even more likely to consider euthanasia as an option for themselves when they did not trust their physicians to follow their end-of-life care wishes [58]. Although these studies seem to indicate that trust does not play the role hypothesized in this study, they have two basic shortcomings: First, they were only concerned with people’s trust in the medical profession or system, and not with their trust in other people in general, or their confidence in monitoring institutions. Second, and most notably, none of these researchers performed a cross-country comparison to account for the potential effects of trust at the country level. However, the mention of high levels of trust in Belgium and the Netherlands might indicate that in the context of euthanasia, trust might be important as a cultural factor, rather than as an individual’s characteristic.

An anecdotal comparison of studies from trust research with studies on end-of-life-attitudes indicates that countries with a liberal attitude toward euthanasia, i.e. Scandinavian countries [19, 49, 59, 60], indeed also exhibit high trust levels (e.g. [61]). In contrast, in countries with low trust, a rather conservative stance toward euthanasia can be observed (e.g. in Eastern Europe).

Also media coverage of end-of-life decisions like euthanasia, assisted suicide, or the forgoing of medical treatment, has reported rather sceptical attitudes in low-trust countries like Italy ([3] p.396), Croatia [62], and Portugal ([63] p.78f). In contrast, in Denmark and Finland, both ranging among the top countries with regard to high trust climates, the media have taken a positive stance on self-determined end-of-life decisions [64, 65]. Similarly, they have proposed a pro-assisted-dying opinion in the medium to high trust countries of Australia [66], the USA [67], Canada [68], Britain [69, 70] and Belgium (c.f., [71]).^c^

To summarize, it might be more important to compare the effect of trust on euthanasia attitudes across countries, rather than just looking for an effect of trust at the level of individuals, as has been done to date.

### The current study

#### Trust objects of interest

The current study gathers new information concerning the role of trust on euthanasia approval among members of the general public. It does so by reporting on the results of a cross-country study analysing the impact of trust on four different actors: 1) the health care system, 2) people in general, 3) state institutions, and 4) the press, and addressing general trust and confidence in health care at the individual level and at the country level.

Trust in the health care system was used, since this perception should reflect the strength of belief that the health care system will provide adequate and affordable treatment [11, 72]. This belief should in turn reduce the fear of involuntary or pushed euthanasia for financial reasons, and lead to more supportive euthanasia attitudes.

Trust in its generalized form, i.e. trusting people in general, including strangers [73], was studied, because involuntary and pushed euthanasia might be caused by a lot of people, or even by the society as a whole; either by acting accordingly in one´s personal sphere or by electing corresponding pro-euthanasia political parties.

As one main purpose of the study was to account for cross-cultural differences, trust in health care and general trust were also included as country indicators. It was assumed that the aggregate perception of trustworthiness and health care adequacy affect euthanasia attitudes beyond the impact of a person’s own level of trust. This might occur, for example, by shaping public debates and media coverage about euthanasia, which is in turn likely to shape individual attitudes [64, 74–76]. To explain, even if an individual does not genuinely think other people are trustworthy, he or she probably derives his or her impression of the permissibility of euthanasia at least partly from public debates, which might, in a high-trust-country, be dominated by assurances of the beneficial aspects of euthanasia, instead of by worries about abuse and social pressures to die (c.f. [77]). A similar indirect effect of public debates has been found for attitudes toward genetically modified food [78], and has also been proposed in the cited 33-country study of Verbakel and Jaspers [19], as an explanation for the fact that personal religiosity and a country´s religious culture impact euthanasia attitudes. Notably, the same study also found euthanasia approval to be higher in countries with a responsive health care system.^d^

Considering other independent variables related to trust, confidence in the already mentioned safeguarding and control mechanisms might be important. Therefore, trust in the state is accounted for, since the state should prohibit the possibility of a slippery slope in which accepted forms of euthanasia transition to unaccepted forms [12]. Similarly, with regard to trust in the state, the perception of the media as being an adequate monitoring institution was analysed, since the media is often understood to be a ‘watchdog’ or ‘fourth estate’, guarding the public interest [13]. Notably, in other (bio)ethical fields, which imply benefits and risks, like genetically modified food, the role of trust in regulators and watchdogs has already been acknowledged as a strong predictor of attitudes towards the issue among the general public [79–81].

Finally, cross-level interactions between the trust variables were considered, since a cultural climate of trust might even increase the effects of a person´s level of trust regarding his or her opinion of euthanasia. A similar cross-level reinforcement effect on euthanasia attitudes has been found for religiosity [19]. Trust at both the individual and the individual country levels have also been found to interact, for example, in affecting health [82, 83].

#### Control variables

The sociodemographic factors of age, education, and gender, as well as religiosity and religious denomination were introduced as control variables, not only because they are generally common control variables, but because they have also frequently been associated with euthanasia attitudes (see above). In addition, values concerning autonomy were controlled for because, as already mentioned, they have been found to affect euthanasia attitudes as well. Autonomy was thought of in three different ways: a) as valuing independence, b) as moral-relativism (c.f. [19]), and c), in terms of an internal locus of control [22] (c.f. [19]). Aggregated responses of moral relativism and postmaterialism [84] were regarded as indicators for values of autonomy at the national level. Postmaterialism was only included as a macro level variable, and not at the individual level, since research indicates that postmaterialist values lack validity at the micro level [85].

Notably, controlling for sociodemographia, religiosity, and various value constructs was additionally important, since these factors have also been associated with trust. For example, moral permissiveness (toward divorce, homosexuality, etc.) and trust are often understood as being part of the broader value construct of postmaterialism or self-expressive values, both being very similar to autonomy values [86]. Controlling for these factors made sure that the posited effects of trust on euthanasia attitudes were really ones of trust, and not of any other underlying phenomenon or confounding factor.

## Methods

### Data and sample

Data describing 49,114 randomly selected individuals in 44 countries were derived from the European Values Study: Wave 4, 2008 [8], hereafter referred to as EVS. The EVS is considered the most comprehensive research project on values that has been conducted in Europe. It is a large-scale, cross-sectional survey research program repeated every nine years, which provides insights into the beliefs, preferences, and attitudes of citizens all over Europe. The EVS is primarily a Social Science survey; ethical approval has not been required. Data are always collected anonymised, and all participants have to give informed consent to data collection. Guidelines about standards and specifications on sampling, fieldwork, data processing and documentation have been developed at Tilburg University in collaboration with CEPS/Instead Luxemburg and the GESIS Data Archive for the Social Sciences in Cologne. The board of the EVS Foundation and National Program Directors in each of the participating countries have assured adherence to the guidelines. For the current purpose, the EVS data were obtained at the GESIS Data Archive for the Social Sciences in Cologne through their online download facilities. For the analyses, data were weighted to ensure the representativeness of the national samples concerning age, gender, and region.^e^

### Measures

#### Dependent variable

Attitudes toward euthanasia were measured with the item ‘Please indicate if euthanasia (terminating the life of the incurably sick) can always be justified, never be justified, or something in between’ (10-point-Likert-scale).

#### Trust measures

General trust was assessed with the commonly used dichotomous question, ‘Generally speaking, would you say that most people can be trusted or that you can’t be too careful dealing with people?’ Answers were coded with 1 indicating a lack of trust (‘you can’t be too careful’), and 2 indicating trust (‘most people can be trusted’). To get national trust rates the answers were recoded to 1, indicating trust, and 0, indicating a lack of trust, and aggregated by country; due to this recoding, the resulting variable can also be interpreted as the percentage of people in each country perceiving others to be trustworthy.

Trust in the health care system was quantified in terms of how much confidence participants had in the health care system of their country of residence (‘Please tell me how much confidence you have in the health care system’). Answers were coded with 1 indicating not at all, 2 indicating not very much, 3 indicating quite a lot, and 4 indicating a great deal. This item was introduced as a quasi-metrical variable. Again, an additional averaged country variable was built by aggregating individual data.

Perceptions regarding the competence and integrity of the press were quantified in the same fashion as trust in the health care system, using a single item.

Trust in the state was captured by four items—confidence in the parliament, the police, the juridical system, and the government—and combined into a single trust-in-the-state-scale (Cronbach’s α = .79).

#### Control variables

Education was assessed in a seven-step categorization process ranging from none or pre-primary education (=0), to second stage tertiary education (=6), and introduced as a continuous variable. As further sociodemographia, age and gender were noted. Religiosity was measured by asking participants how important religion was for them, with 0 indicating not at all important, 1 indicating not important, 2 indicating quite important, and 3 indicating very important. Answers were aggregated to the country level, to get a variable for the level of religiosity within a country. Questions concerning religious denomination allowed participants to choose between ‘no denomination’ (which served as a reference category in the following analysis), Roman Catholic, Protestant, Free Church/Evangelical, Jewish, Muslim, Hindu, Buddhist, Orthodox, or others. Since very few participants described themselves as Hindu or Buddhist, both denominations were grouped together with ‘other denomination’. To control for denominational context at the country level, the percentage of people of Protestant denomination was used. Protestantism was chosen since Protestant cultures are linked to higher general levels of trust [61], and therefore—paralleling the inclusion of value constructs—using Protestantism should also ensure that the impact of trust is not the impact of a broader cultural construct.

Regarding appreciation and feelings of autonomy, autonomy as valuing independence was measured in the form of a dummy variable, by whether participants chose independence as a quality children should be encouraged to learn, out of a list of eleven qualities. Moral-relativism was assessed by asking whether participants thought there are absolute, universal guidelines (coded 1), absolute, universal guidelines with exceptions (coded 2), or no absolute, universal guidelines (coded 3). Locus of control was measured by asking participants how much freedom of choice and control they feel they have over the way their life turns out, using a 10-point-Likert-scale. One indicated none at all, and 10 indicated a great deal. Finally, postmaterialism was measured by asking individuals which of the following country aims is most important to them: (1) maintaining order in the nation, (2) giving the people more say in important government decisions, (3) fighting rising inflation, or (4) protecting freedom of speech. Respondents who chose responses 1 and 3 were classified as materialists; those who chose responses 2 and 4 were classified as postmaterialists, and the remaining combinations were classified as mixed. The final country-level-variable was developed by calculating the proportion of postmaterialists in each of the countries.

## Method

Data were introduced into a multilevel analysis using SPSS mixed models. Multilevel analyses in general are advisable when there are two (or more) levels of analysis, with one level (here individual people) nested in level two aggregates (here countries). Using this research method allows us to exclude the variability between higher units (countries) when observing the variability of subordinate levels (individual people). Therefore it leads to more accurate results, when independent variables are to be analysed at both the individual and the country level (for a detailed description of multilevel analysis see [87]).

As recommended for multilevel models [87], country level variables were mean-centred for the regression analysis, that is, the regression intercepts show the value for a country with average levels of trust, confidence in health care, religiosity, Protestantism, moral relativism and postmaterialism, and the regression coefficients of the five variables indicate the increase or decrease relative to this hypothetical mean country. Indicators at the individual level were not centred, to make the trust/not-trust-dummy-variable easier to interpret.

Different models were tested by adding further variables to each former model, using a stepwise method (see below).^f^

## Results

### Descriptive analysis

**Figure 1:**
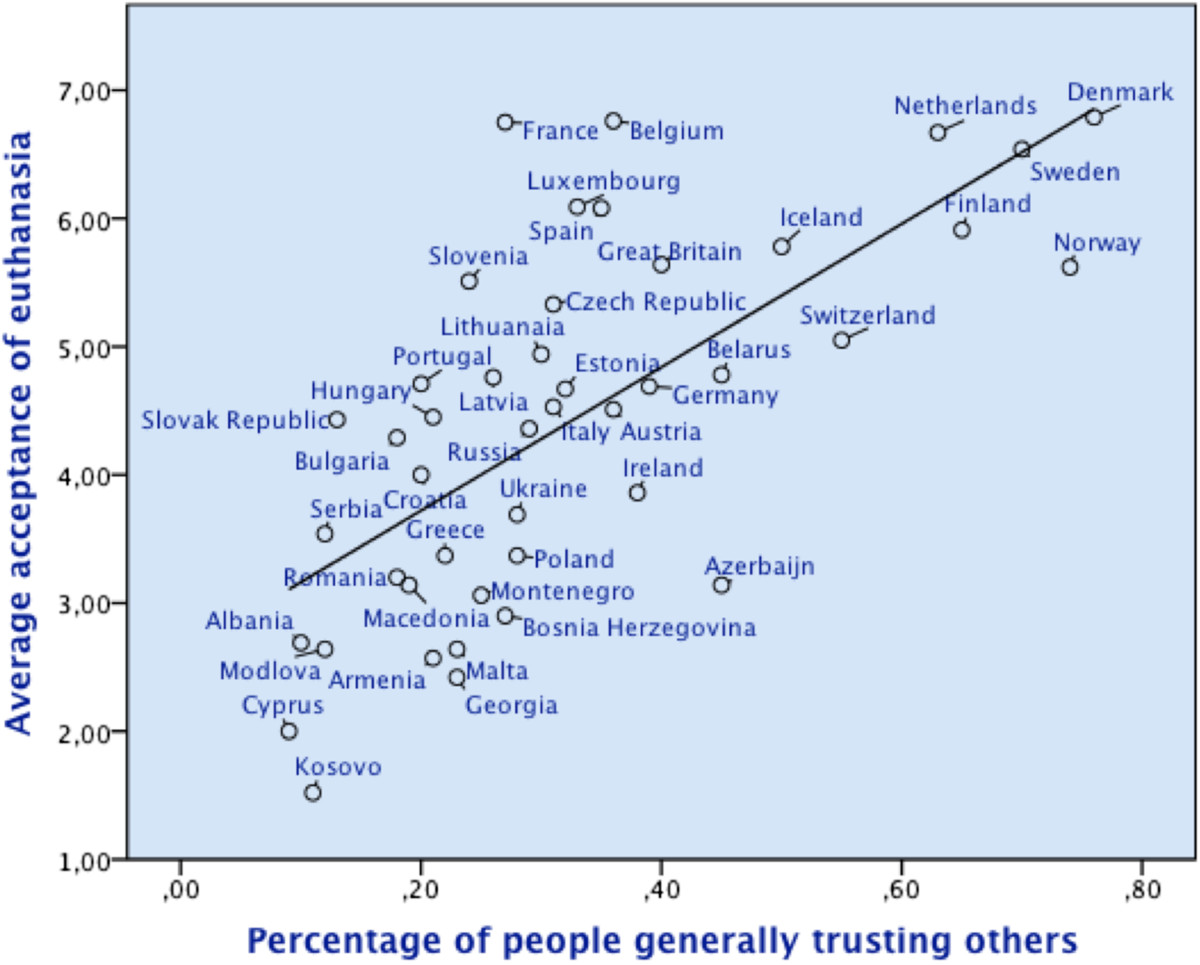
**Average acceptance of euthanasia and trust rates by country.** The scatter plot and the corresponding regression line show that high trust countries usually exhibit also a rather high acceptance of euthanasia, while citizens in low trust countries have a more restrictive euthanasia attitude. Data source: European Values Study, 2008.

On average, participants indicated a euthanasia acceptance value of 4.50 (SD = 3.22), thereby being at mean, slightly against, rather than in favour of euthanasia. However, attitudes varied widely between individuals, as well as between countries (see Tables 1 and 2). The highest approval ratings for euthanasia could be observed in Denmark (M = 6.79; SD = 2.84) and Belgium (M = 6.76; SD = 2.60), with similar high levels of acceptance in the other Benelux and Scandinavian countries. The lowest levels of permissiveness were reported in Kosovo (M = 1.52; SD = 1.67) and Cyprus (M = 2.00; SD = 2.05), with comparably low rates in other Eastern-Mediterranean and Balkan countries.Similar to euthanasia attitudes, levels of trust varied, with the Eastern Mediterranean and Balkan countries, and the Scandinavian/Benelux countries again presenting the most extreme cases. For example, in Cyprus only 9 per cent (SD = .29), and in Kosovo only 11 per cent (SD = .31) of all participants thought other people in general were trustworthy, which contrasted sharply with 76 per cent of the people in Denmark trusting other people (SD = .43). Trust in the health care system was at a medium to high level overall (M = 2.63; SD = .85). Nevertheless, again it was highest in countries like Belgium (M = 3.23, SD = 62) and Luxemburg (M = 3.22, SD = .72), while being relatively low in Eastern European countries like Bulgaria (M = 1.85; SD = .72). As Figure 1 shows, euthanasia acceptance and levels of trust have a remarkably similar distribution across countries, with those showing high levels of trust often also being quite permissive concerning euthanasia.

**Table 1:** **Descriptiva for variables of main interest**

	Euthanasia acceptance (possible range 1–10)	General trust (possible range 0–1)	Confidence in health care system (possible range 1–4)	Confidence in state scale (possible range 1–4)	Confidence in press (possible range 1–4)	General trust Country aggregate (possible range 0–1)	Health care confidence country aggregate (possible range 1–4)
Mean (SD)	4.39 (3.22)	.31 (.46)	2.61 (.86)	2.38 (.68)	2.26 (.80)	.31 (.16)	2.61 (.32)

Table legend: Means and standard deviations of euthanasia attitude and trust items.

Data source: European Values Study, 2008. Missing data were dealt with using pairwise deletion.

**Table 2:** **Euthansia acceptance, general trust and trust in health care by country; Mean (SD)**

	Euthanasia acceptance (possible range 1–10)	Trust rates (possible range 0–1)	Trust in health care system (possible range 1–4)
Albania	2.69 (2.54)	.10 (.31)	2.07 (.81)
Armenia	2.57 (2.43)	.21 (.41)	2.68 (.41)
Austria	4.51 (3.07)	.36 (.48)	2.88 (.78)
Azerbaijan	3.14 (2.80)	.45 (.50)	2.81 (.93)
Belarus	4.78 (2.81)	.45 (.50)	2.72 (.72)
Belgium	6.76 (2.60)	.36 (.48)	3.23 (.62)
Bosnia Herzegovina	2.90 (2.80)	.27 (.44)	2.66 (.87)
Bulgaria	4.29 (3.13)	.18 (.39)	1.85 (.85)
Croatia	4.06 (3.16)	.20 (.40)	2.35 (.76)
Cyprus	2.00 (2.05)	.09 (.29)	2.61 (.86)
Czech Republic	5.33 (2.95)	.31 (.46)	2.34 (.83)
Denmark	6.79 (2.84)	.76 (.43)	2.88 (.66)
Estonia	4.67 (2.87)	.32 (.47)	2.50 (.75)
Finland	5.91 (2.88)	.65 (.48)	2.81 (.67)
France	6.75 (2.80)	.27 (.45)	2.93 (.70)
Georgia	2.42 (2.21)	.23 (.42)	2.58 (.85)
Germany	4.69 (2.94)	.39 (.49)	2.24 (.85)
Greece	3.37 (2.78)	.22 (.41)	2.24 (.84)
Hungary	4.45 (3.24)	.21 (.41)	2.17 (.80)
Iceland	5.78 (2.87)	.50 (.50)	3.31 (.59)
Ireland	3.86 (2.79)	.38 (.49)	2.12 (.87)
Italy	4.53 (3.24)	.31 (.46)	2.54 (.79)
Kosovo	1.52 (1.67)	.11 (.31)	2.91 (.88)
Latvia	4.76 (3.05)	.26 (.44)	2.51 (.81)
Lithuania	4.94 (2.98)	.30 (.46)	2.19 (.66)
Luxembourg	6.09 (3.35)	.33 (.47)	3.22 (.72)
Macedonia	3.14 (2.80)	.19 (.40)	2.51 (.86)
Malta	2.64 (2.70)	.23 (.42)	3.09 (.76)
Moldova	2.64 (2.61)	.12 (.33)	2.52 (.79)
Montenegro	3.06 (2.97)	.25 (.44)	2.58 (.88)
Netherlands	6.67 (2.79)	.63 (.48)	2.79 (.68)
Norway	5.62 (2.91)	.74 (.44)	2.86 (.68)
Poland	3.37 (2.75)	.28 (.45)	2.34 (.86)
Portugal	4.71 (3.00))	.20 (.40)	2.54 (.78)
Romania	3.20 (2.84)	.18 (.38)	2.53 (.90)
Russia	4.36 (3.11)	.29 (.46)	2.44 (.85)
Serbia	3.54 (3.15)	.12 (.33)	2.34 (.82)
Slovak Republic	4.43 (3.13)	.13 (.33)	2.55 (.75)
Slovenia	5.51 (3.28)	.24 (.43)	2.71 (.67)
Spain	6.08 (3.20)	.35 (.48)	2.96 (.74)
Sweden	6.54 (2.90)	.70 (.46)	2.85 (.69)
Switzerland	5.05 (3.11)	.55 (.50)	2.95 (.69)
Ukraine	3.69 (3.20)	.28 (.45)	2.29 (.91)
United Kingdom	5.64 (3.06)	.40 (.49)	3.04 (.76)

Table legend: Country means and standard deviations for euthanasia attitude, general trust and confidence in health care by country.

Data source: European Values Study, 2008. Missing data were dealt with using pairwise deletion.

### Multilevel regression

The multilevel analysis tested five different models (see Table 3). In model 1, only individual level variables of interest were introduced (general trust, confidence in the state, the health care system, and the press). The effects of general trust and trust in the press were significant and in the assumed direction: Participants who thought most others could be trusted rated euthanasia to be more justifiable than did participants who felt that one cannot be too careful concerning other people (B = .14, p < .01). Similarly, participants believing in the competence of the press showed slightly more favourable euthanasia attitudes (B = .09, p < .01). However, in contrast to the study’s hypotheses, euthanasia acceptance decreased slightly, the more participants perceived the health care system to be reliable (B = −.05, p < .01). The same counter-hypothesized trend held for confidence in the state, which was negatively related to euthanasia attitudes as well (B = −.58, p < .01).

**Table 3:** **Multievel-regression on individual level euthanasia acceptance**

Independent variable	Null model	Modell 1 (individual-level trust variables)	Model 2 (model 1 plus sociodemographic control variables)	Model 3 (Model 2 plus control for autonomy values)	Model 4 (model 3 plus country level trust	Model 5 (model 4 plus country level control variables)
Intercept		5.71** (23)	7.10** (20)	5.39** (.20)	6.35** (.15)	6.37** (.13)
Individual level variables		.14** (.03)	.06* (.03)	.06* (.03)	.06* (.03)	.06* (.03)
General trust						
Reference: not trusting						
Confidence in health care system		- .05** (.02)	- .03 (.02)	- .03 (.02)	- .03 (.02)	- .03 (.02)
Confidence in press		.09** (.02)	.07** (.02)	.07** (.02)	.07** (.02)	.07** (.02)
Confidence in state scale		- .58** (.03)	-. 43** (.03)	- .42** (.03)	- .42** (.03)	- .42** (.03)
Age			- .01** (.00)	- .01** (.00)	- .01** (.00)	- .01** (.00)
Education			.14** (.01)	.13** (.01)	.13** (.01)	.13.** (.01)
Gender male			- .01 (.03)	- .01 (.03)	- .01 (.03)	- .01 (.03)
Reference female						
Religiousness			- .57** (.02)	- .55** (.02)	- .55** (.02)	- .55** (.02)
Religious denomination:						
Catholic			- .30** (.04)	- .27** (.04)	- .27** (.04)	- .27** (.04)
Muslim			- .79** (.07)	- .76** (.07)	- .76** (.07)	- .76** (.07)
Jewish			−1.05** (.32)	−1.02** (.32)	−1.02** (.32)	−1.02** (.32)
Protestant Free Church			−1.21** (.18)	−1.16** (.18)	−1.16** (.18)	−1.16** (.18)
Orthodox			- .05 (.05)	- .04 (.05)	- .04 (.05)	- .03 (.05)
Protestant			- .05 (.06)	- .05 (.06)	- .06 (.06)	- .06 (.06)
Other denomination			- .62** (.10)	- .56** (.10)	- .56** (.10)	- .56** (.10)
Reference: non-denominational						
Valuing independence				.26** (.03)	.26** (.03)	.26** (.03)
Reference: not mentioned						
Internal locus of control				.03** (.01)	.03** (.01)	.03** (.01)
Moral relativism						
Moral relativism				.48** (.03)	.48** (.03)	.48** (.03)
Moral exceptionalism				.34** (.03)	.34** (.03)	.34** (.03)
Reference: moral absolutism						
Country level variables					3.82** (.81)	2.36* (0.98)
General trust						
Health care confidence					1.00* (.42)	1.09** (.32)
Religiousness						−1.09** (.24)
Denominational share protestants						- 1.85* (.69)
Moral relativism country mean						1.23* (.56)
Postmaterialism						1.15 (.74)
Covariance Parameters						
Residual (individual level variance)	8.35** (.05)	8.22** (.05)	7.73** (.05)	7.67** (.05)	7.67** (.05)	7.67** (0.5)
Intercept (country level variance)	2.01** (.43)	2.10** (.46)	1.46** (.33)	1.36** (.31)	.68** (.15)	.33** (.08)
Model Fit Parameters	-					
Variance explained at the individual level (compared to null model)		1.68%	7.43%	8.14%	8.14%	8.14%
Variance explained at the country level (compared to null model)	-	0.00%	27.36%	32.34%	66.17%	83.85%
AIC	-	253955.181	250792.121	250460.903	250427,692	250393,525
BIC	-	253972.872	250809.811	250478.593	250445,382	250411,215
Deviance (−2LL)	-	253951.181	250788.121	250456,903	250423.692	250389,525
X^2^(df) (comparison with previous model)	-	X^2^ (4) =774.63 **	X^2^(4) =3162.06 **	X^2^(3) =331.22 **	X^2^(2) =33,21**	X^2^(4) =34.17 **

Unstandardized coefficients with standard errors in parentheses.

Table legend: Data source: European Values Study, 2008, N = 49.114.

Variables of main interest in bold. **significance at 1% level; * significance at 5% level.

Model 2 took account of the sociodemographic control variables, i.e. age, education, and gender, as well as religiousness and religious denomination. As in other studies, euthanasia acceptance decreased with age, although only slightly (B = −.01, p < .01), while it increased with education (B = .14, p < .01). No differences were found due to gender. Also mirroring prior studies, the more religious participants said they were, the less permissible they perceived euthanasia to be (B = −.57, p < .01). An additional effect emerged for denomination, such that self-defined Catholic, Muslim, Jewish, and Free Church/Evangelical participants were less supportive of euthanasia than non-denomination participants, with no differences evident for Protestants, and surprisingly also not for Orthodox believers (for actual coefficients see Table 3). Despite the control variables, the trust variables stayed substantially the same, and decreased only slightly in magnitude. The only difference was trust in health care, which lost significance.

Model 3 also included the additional control variables of autonomy values. All three autonomy variables pointed in the expected direction. People explicitly valuing independence were more supportive of euthanasia than participants who did not (B = .26, p < .01). Similarly, euthanasia acceptance increased slightly with the perception of an internal locus of control (B = .03, p < .01), and also with viewing moral guidelines not as absolute and universal, but rather as relative (B = .48, p < .01), or at least as allowing for exceptions (B = .34, p < .01). Again, general trust, trust in the press, and confidence in the state did not lose significance, despite the control variables.

In the next step, model 4 also included the two country characteristics of primary interest, namely country level general trust and confidence in the health care system. Both trust rates at the country level showed a pronounced effect beyond individual’s trust levels for both general trust (B = 3.82, p < .01) and confidence in the health care system (B = 1.00, p < .05). The latter was notably the case, despite the initially contradictory negative impact of health care trust on the individual level.

Model 5 also showed that other country characteristics like religiosity (B = −1.09, p < .01), denominational context (B = −1.85, p < .05), and moral relativism (B = 1.23, p < .05) predicted euthanasia attitudes, only postmaterialism did not add further explanation. Still, all trust variables held up to give direction to euthanasia acceptance. The impact of country level confidence in health care even increased.

Finally, cross-level interactions between the trust indicators were tested (see Table 4). It turned out that a cultural climate of general trust was less important for generally trusting individuals (B = −.35, p < .05). Turned the other way around, general trust at the individual level only had a positive effect on euthanasia attitudes in countries with average or below average levels of general trust, but it had a negative effect in countries with above average general trust levels. A cultural climate of general trust similarly interacted with individual’s levels of confidence in the health care system, such that the main effect of trust in health care, i.e. the effect in countries with an average level of general trust, was still insignificant, but in countries with above average general trust, confidence in health care decreased euthanasia acceptance (B = −.31, p < .01). Finally, the contra-intuitive negative impact of confidence in the state was increased in countries with above average general trust (B = −.57, p < .01).

**Table 4:** **Significant cross-level interactions**

	General trust x country level trust	Confidence in health care x country level trust	Confidence in state x country level trust
Main effect individual Level	07* (.03)	-.03 (.02)	-.43** (.03)
Main effect country level	2.50* (.98)	3.17** (1.01)	3.82** (1.04)
Cross-level interaction	-.35* (.18)	-.31** (.10)	-.57** (.13)

Unstandardized coefficients with standard errors in parentheses.

Table legend: Data source: European Values Study, 2008.

**significance at the 1% level; * significance at the 5% level.

Turning to the explanatory significance of the models, it should be noted in advance that assessing absolute model fits and explanatory powers in multilevel models is much more complicated than in single-level regressions, since the commonly used R-square statistic cannot be applied directly because variance in the dependent variable can originate from variance between level-one units (individual people) as also from variance between level-two units (countries) (for a detailed discussion see 87). Therefore, the first thing to do is to analyse how much of the variance is attributed to which of the levels. This is done by looking at the so-called null model (the model with no independent variables only accounting for variation between countries), and calculating so called interclass correlation coefficients (ICC), which state how much of the variance in the dependent variable of interest is caused by variance between level two units, rather than by variance between level one units (individuals). In the current dataset it turned out that overall 19.4 per cent of the variance in euthanasia attitudes could be attributed to between-country difference; meaning the attitude toward euthanasia differed more between individual people within countries than between countries, but to a considerable extent a person’s euthanasia attitude is influenced by the country he or she lives in.

Knowing this, the final model (model 5) explained about eight per cent of the within-country variance in euthanasia attitudes, and 84 per cent of the between-country variance. Comparing model 3 with the null model furthermore showed that about one third of the initial between-country differences in euthanasia attitudes were explained by the individual level variables included (model 3), and, as such, were compositional effects rather than real country characteristics. Very important for the study was the finding that individual level trust variables (model 1) accounted for only about 2 per cent of within-country differences in euthanasia attitudes, thereby having rather little impact. However, the two country level trust variables introduced in model 4 turned out to be highly relevant for explaining between-country differences in euthanasia acceptance, since they explained half of the between-country variance which had remained after the inclusion of all individual level variables (model 3). Further model fits for comparing the different models like deviance and Akaike’s Information Criterion (AIC) can be derived from Table 3. These criteria should not be interpreted absolutely, but only for the purpose of selecting the model that fits best by searching for the lowest values. As can be seen, independent of the criterion used, each model is able to improve the data fit.

## Discussion

This study has helped to answer the question of what leads people to either favour or oppose euthanasia, by showing that trust at the individual level, and especially at the country level, is linked to a more permissive euthanasia attitude. By analysing data from over 40,000 individuals in 44 countries, it was shown that, as hypothesized, people indicate that they have more favourable opinions on euthanasia when they believe that most other people can be trusted, and when they have confidence in the press. At the country level, high levels of trust in other people, as well as high levels of confidence in the health care system, increased euthanasia acceptance. This response held beyond individual level trust, i.e. even if a person did not think others could be trusted and was not confident about the health care system, his or her attitude toward euthanasia was more favourable when he or she lived in a country where many other people were trustful and confident. The pronounced effect of country level trust is consistent with previous research findings, which have shown acceptance of euthanasia to be higher in countries with responsive health care systems [19], and to be strongly influenced by country characteristics in general (e.g. [49, 88, 89]).

While the effects of trust at the country levels, and of confidence in the press at the individual level, supported the proposed hypotheses, the findings with regard to trust in the state, and in health care at the individual level, did not, since they reduced, rather than increased, the acceptance of euthanasia.

The seeming paradox that people who do not put much confidence in the state are more lenient towards euthanasia might in turn be due to problems of operationalization. As other authors noted, asking for ‘confidence in the state’ might not measure an estimate of the state’s competence, but rather provide a measure of the extent to which the state is being rejected as an authority [86]. Given that euthanasia is illegal in most countries, both rejecting the state´s authority and supporting euthanasia may be caused by protest proneness, which might not have been captured by the autonomy control variables included.

To explain the negative effect of individual level confidence in the health care system is less straightforward. The negative effect was contrary to the reasoning provided in the introduction since trust in the health care system was assumed to increase acceptance of euthanasia. However, it might indeed also be reasonable that trust decreases euthanasia acceptance. To clarify, on the one hand it was assumed that trust increases euthanasia acceptance, because trust should render fear of abuses less likely. On the other hand believing the health care system does *not* provide adequate care might as well shed a more positive light on euthanasia, as euthanasia might become more desirable in times of serious illness, if no adequate health care is available (c.f. [90]). This connection is one that some proponents of palliative care have also been drawing, namely those who expect better palliative care (as part of the health care system) to render the need for euthanasia less necessary [91]. Both effects have been found in the current study—a cultural climate of confidence in health care was related to more permissive euthanasia attitudes, but regarding differences between individual people, low confidence was related to slightly more permissive euthanasia attitudes (notably after having removed cross-country differences from the individual level due to using multilevel analysis). Hence, it seems that confidence in the health care system plays a different role at the country level than at the individual level. As already suggested in the introduction, a cultural climate of trust might shape euthanasia attitudes rather unconsciously through public debates, with the media paying no heed to risks and the potential slippery slope, so that those risks are not salient to the citizen. At the individual level however, the slightly negative effect of heath care confidence on euthanasia attitudes might rather be attributable to conscious perceptions of health care confidence. Thereby, the findings of the current study do also not contradict the studies mentioned in the introduction, which found either no effect or a negative effect of trust on euthanasia [55–57] since those have only considered confidence in the health care system as an individual person´s characteristic.

### Limitations and future studies

The basic shortcoming of this study is that the mechanisms behind the effects of trust on euthanasia attitudes could not be examined using the current dataset. It has been assumed that having trust affects attitudes towards euthanasia by reducing slippery slope fears, i.e. the fear of euthanasia abuses and expansions or even involuntary deaths [20]. However, no indicators of slippery slope fears had been included in the dataset used. Future research might be strongly encouraged to clarify the relationship between euthanasia attitudes and trust by having a look at the cause-effect direction, and testing whether the impact of trust on euthanasia attitudes is really mediated by slippery slope fears.

Concerning the strong country effect of general trust, detailed consideration might also be given to potential explanations in light of social capital theory, since trust can also be understood as a constituent part—or at least a very closely linked consequence or antecedent—of social capital [92], and high levels of trust and social capital at the country or community level have been shown to go together with solidarity between people [93] and to decrease crime, and fear of crime [94–96].

Similarly, further studies should clarify why confidence in the health care system has a different effect at the country level and at the individual level, such that people living in countries with a culture of high health care trust have much more positive attitudes toward euthanasia, while within the countries people who have confidence in the health care system are less in favour of euthanasia acceptance than people with less confidence, and whether the effect at the country level is indeed moderated by the valence of media reports and public debates as considered in the introduction. Ideally, a longitudinal study should be conducted to shed some more light on the causal relationships.

Notably, a longitudinal study would also be worthwhile for looking at possible trust-euthanasia-loops, in terms of thinking not only of the effect of trust on euthanasia acceptance, but also the effect of euthanasia practices on trust. This reversed relationship would seem interesting as well, since prior to the current study the euthanasia-trust-debate had been pursued on exactly that reversed relationship, i.e. on potential decreases in trust (especially regarding trust in physicians) because of euthanasia [97–99]. The (still) high levels of trust in the health care system in Belgium, Luxemburg, and the Netherlands might suggest that legalisation has not eroded trust. However, social climates of trust need a long time to change [61]. It would be a worthwhile, although lengthy, endeavour to clarify how trust and euthanasia attitudes and practice, respectively, shape each other, and whether the potential negative, positive, or non-existing effect of legal euthanasia on trust depends on the initial levels of trust.

Further suggestions for future studies include performing a cross-cultural study with the inclusion of trust in physicians [100], and replicating the findings of this paper using more sophisticated measures of trust and euthanasia attitudes than the abstract one-item measures included in the EVS (e.g. [101, 102] c.f. [103]). In addition, future studies might differentiate between the moral justification for euthanasia (as included in this study), and attitudes toward its legalisation, since the latter should matter even more in the slippery slope context.

### Possible normative implications

This article has taken a socio-empirical approach to a normative-philosophical issue (c.f. [104, 105]. Despite the currently intensive normative debate about end-of-life issues, the paper is not supposed to take any position on the question as to whether euthanasia should be legalised or not. However, it is quite undisputed that slippery slope fears lead to ‘inefficiencies’ [24]. In the case of euthanasia, adhering to the contra-argument of possible abuses leads to prohibiting euthanasia in general—and therefore also to cases in which most people would consider euthanasia to be reasonable. Since uninformed judgments of others´ trustworthiness are often erroneous [106, 107], this paper might encourage reflection on the question of whether attitudes toward trust or distrust regarding euthanasia are well grounded or not, and to adopt attitudes and policies accordingly.

## Conclusion

By showing that trust and a cultural climate of trust go hand in hand with permissive euthanasia attitudes, this paper concludes that trust seems to be a noteworthy construct to explain differences in attitudes towards euthanasia, especially when drawing cross-country comparisons. Whether the effect of trust is really mediated by the counteracting of slippery slope fears must be considered in future studies. In any case, this paper hopes to enrich theoretical and empirical encounters during end-of-life-decisions, and supposes that trust should be added to the existing literature of correlates of euthanasia attitudes.

### End notes

^a^Slippery slope arguments can arise in two ways—a logical and a practical form [20, 24, 35]. The logical argument refers to the assumption that allowing for an act A (one kind of euthanasia, respectively) will lead to allowing for another act B (or kind of euthanasia) as well, because A and B are theoretically not different, or the justification for A might also apply to B. (c.f. [108–110]). The practical slippery slope argument—also called the empirical or psychological slippery slope argument—in contrast, states that acceptable and unacceptable forms of euthanasia (A and B, respectively) are logically distinguishable, but might nevertheless merge in practice.

^b^Notably, different levels of trust have also been shown to explain cross-country differences in attitudes towards other bioethical fields, which imply benefits and risks, such as biotechnology [111].

^c^For the Netherlands, the media tenor had been described as being very uncritical of euthanasia [112] although this seems to have changed recently [113].

^d^Health care system responsiveness has been defined by the World Health Organization (WHO) as respect for the dignity of the person, autonomy to participate in health decision-making, quality of care, and confidentiality [114].

^e^The EVS 2011 included 47 countries with 67,786 participants, but applying listwise deletion of missing values led to a reduced number of participants, and to the exclusion of Northern Cyprus, Northern Ireland, and Turkey.

^f^Notably, many of the independent variables might have been correlated, which could have biased the results, but including all variables seemed theoretically appropriate. Furthermore, multicollinearity was not an empirical problem, since the variance inflation factor (VIF) ranged from 1.02 to a maximum of 3.90, thereby being below any critical value [115]. The VIF gives evidence of the magnitude of collinearity by analysing how much of the variance in one independent variable can be explained by the other independent variables, and how far this inflates the standard error of that variable’s regression coefficient. It is disputable from which factor onwards collinearity is problematic, but usually only VIF higher than 10 are considered critical [115].

## Authors’ original submitted files for images

Below are the links to the authors’ original submitted files for images. Authors’ original file for figure 1 Authors’ original file for figure 2

## References

[1] Christie B (2013). New bid to legalise assisted suicide reaches Scottish parliament. BMJ.

[2] Delamothe T, Snow R, Godlee F (2014). Why the assisted dying bill should become law in England and Wales. BMJ.

[3] Griffiths J, Weyers H, Adams M (2008). Euthanasia and law in Europe.

[4] Kermode-Scott B (2013). Euthanasia and aiding suicide to remain criminal acts in Canada. BMJ.

[5] Spranzi M (2013). The French euthanasia debate: exception and solidarity. Camb Q Healthc Ethics.

[6] Teisseyre N, Mullet E, Sorum PC (2005). Under what conditions is euthanasia acceptable to lay people and health professionals?. Soc Sci Med.

[7] Smith WJ (2003). Forced Exit: The Slippery Slope from Assisted Suicide to Legalized Murder.

[8] EVS (2011). European Values Study 2008: Integrated Dataset.

[9] Cook K (2001). Trust in society.

[10] Delhey J, Newton K (2005). Predicting cross-national levels of social trust: global pattern or Nordic exceptionalism?. Eur Sociol Rev.

[11] Battin MP (1987). Age rationing and the just distribution of health care: Is there a duty to die?. Ethics.

[12] Smets T, Bilsen J, Cohen J, Rurup ML, De Keyser E, Deliens L (2009). The medical practice of euthanasia in Belgium and The Netherlands: legal notification, control and evaluation procedures. Health Policy.

[13] Demers D, Viswanath K (1999). Mass Media, Social Control, and Social Change: A Macrosocial Perspective.

[14] Emanuel EJ (1994). The history of euthanasia debates in the United States and Britain. Ann Intern Med.

[15] YouGov UK (2013). Dignity in Dying Survey Results.

[16] Hendry M, Pasterfield D, Lewis R, Carter B, Hodgson D, Wilkinson C (2013). Why do we want the right to die? A systematic review of the international literature on the views of patients, carers and the public on assisted dying. Palliat Med.

[17] Rietjens JA, van der Heide A, Onwuteaka-Philipsen BD, van der Maas PJ, van der Wal G (2006). Preferences of the Dutch general public for a good death and associations with attitudes towards end-of-life decision-making. Palliat Med.

[18] Kemmelmeier M, Wieczorkowska G, Erb HP, Burnstein E (2002). Individualism, authoritarianism, and attitudes toward assisted death: cross-cultural, cross-regional, and experimental evidence. J Appl Soc Psychol.

[19] Verbakel E, Jaspers E (2010). A comparative study on permissiveness toward Euthanasia. Public Opin Q.

[20] van der Burg W (2009). The slippery-slope argument.

[21] Kohlberg L, Hersh RH (1977). Moral development: a review of the theory. Theory Pract.

[22] Rotter JB (1966). Generalized expectancies for internal versus external control of reinforcement. Psychol Monogr.

[23] Walton DN (1992). Slippery Slope Arguments.

[24] Volokh E (2003). The mechanisms of the slippery slope. Harv Law Rev.

[25] Smith SW (2005). Fallacies of the logical slippery slope in the debate on physician-assisted suicide and euthanasia. Med Law Rev.

[26] Burgess JA (1993). The great slippery-slope argument. J Med Ethics.

[27] O'Mathuna DP (2006). Human dignity in the Nazi era: implications for contemporary bioethics. BMC Med Ethics.

[28] Hardwig J, Denier Y, Gastmans C, Vandevelde A (2014). Is there a duty to die in Europe? If not now, when?. Justice, luck & responsibility in health care.

[29] Rietjens JA, van der Heide A, Onwuteaka-Philipsen BD, van der Maas PJ, van der Wal G (2005). A comparison of attitudes towards end-of-life decisions: survey among the Dutch general public and physicians. Soc Sci Med.

[30] Giordano S (2010). Anorexia and refusal of life-saving treatment: the moral place of competence, suffering, and the family. Philos Psychiatr Psychol.

[31] Gather J, Vollmann J (2013). Physician-assisted suicide of patients with dementia. A medical ethical analysis with a special focus on patient autonomy. Int J Law Psychiatry.

[32] Keown J (2002). Euthanasia, ethics and public policy: an argument against legalisation.

[33] Callahan D (1992). When Self‒Detertnination Runs Amok. Hastings Cent Rep.

[34] Lamb D (1988). Down the Slippery Slope: Arguing in Applied Ethics.

[35] Lewis P (2007). The empirical slippery slope from voluntary to non-voluntary euthanasia. J Law Med Ethics.

[36] Rietjens JA, van der Maas PJ, Onwuteaka-Philipsen BD, van Delden JJ, van der Heide A (2009). Two decades of research on euthanasia from the Netherlands. What have we learnt and what questions remain?. J Bioeth Inq.

[37] Smets T, Bilsen J, Cohen J, Rurup ML, Deliens L (2010). Legal euthanasia in Belgium: characteristics of all reported euthanasia cases. Med Care.

[38] Grand-Duché de Luxembourg: Loi sur l’euthanasie et l’assistance au suicide.http://www.legilux.public.lu/leg/a/archives/2009/0046/a046.pdf,

[39] Buiting H, van Delden J, Onwuteaka-Philpsen B, Rietjens J, Rurup M, van Tol D, Gevers J, van der Maas P, van der Heide A (2009). Reporting of euthanasia and physician-assisted suicide in the Netherlands: descriptive study. BMC Med Ethics.

[40] Pereira J (2011). Legalizing euthanasia or assisted suicide: the illusion of safeguards and controls. Curr Oncol.

[41] Costa AC, Bijlsma-Frankema K (2007). Trust and control interrelations new perspectives on the trust—control nexus. Group Organ Manage.

[42] Shapiro SP (1987). The social control of impersonal trust. AJS.

[43] O'Neill O (2002). Autonomy and trust in bioethics.

[44] Enoch D (2001). Once you start using slippery slope arguments, you're on a very slippery slope. OJLS.

[45] Cohen-Almagor R (2002). Why The Netherlands?. J Law Med Ethics.

[46] Trappenburg M, Oversloot H, Yougner SJ, Kimsma GK (2012). The Dutch Social Fabric Health Care, Trust, and Solidarity. Physician-Assisted Death in Perspective: Assessing the Dutch Experience.

[47] Andrew EV, Cohen J, Evans N, Menaca A, Harding R, Higginson I, Pool R, Gysels M, Prisma (2013). Social-cultural factors in end-of-life care in Belgium: a scoping of the research literature. Palliat Med.

[48] Bernheim JL, Distelmans W, Mullie A, Ashby MA (2014). Questions and answers on the Belgian model of integral end-of-life care: experiment? Prototype?. J Bioeth Inq.

[49] Cohen J, Van Landeghem P, Carpentier N, Deliens L (2013). Different trends in euthanasia acceptance across Europe. A study of 13 western and 10 central and eastern European countries, 1981–2008. Eur J Public Health.

[50] Battin M (1992). Voluntary euthanasia and the risks of abuse: can we learn anything from The Netherlands?. Law Med Health Care.

[51] Yücel Ö, Heijnen A (2014). Assisted dying in Turkey. EAMCE Newsletter 36.

[52] Wasserman J, Clair JM, Ritchey FJ (2006). Racial differences in attitudes toward euthanasia. OMEGA-J Death Dying.

[53] Braun KL, Tanji VM, Heck R (2001). Support for physician-assisted suicide: exploring the impact of ethnicity and attitudes toward planning for death. Gerontologist.

[54] McKinley ED, Garrett JM, Evans AT, Danis M (1996). Differences in end-of-life decision making among black and white ambulatory cancer patients. J Gen Intern Med.

[55] Ward RA (1980). Age and acceptance of euthanasia. J Gerontol.

[56] Clery EMS, Philips M, Park A, Curtis J, Thomson K, Phillips M, Johnson M (2007). Quickening death: the euthanasia debate. British Social Attitudes: the 23rd Report – Perspectives on a changing society.

[57] Raijmakers NJ, van der Heide A, Kouwenhoven PS, van Thiel GJ, van Delden JJ, Rietjens JA (2013). Assistance in dying for older people without a serious medical condition who have a wish to die: a national cross-sectional survey. J Med Ethics.

[58] Buiting HM, Deeg DJ, Knol DL, Ziegelmann JP, Pasman HR, Widdershoven GA, Onwuteaka-Philipsen BD (2012). Older peoples' attitudes towards euthanasia and an end-of-life pill in The Netherlands: 2001–2009. J Med Ethics.

[59] Cohen J, Marcoux I, Bilsen J, Deboosere P, van der Wal G, Deliens L (2006). European public acceptance of euthanasia: socio-demographic and cultural factors associated with the acceptance of euthanasia in 33 European countries. Soc Sci Med.

[60] Cohen J, Van Landeghem P, Carpentier N, Deliens L (2014). Public acceptance of euthanasia in Europe: a survey study in 47 countries. Int J Public Health.

[61] Bjørnskov C (2007). Determinants of generalized trust: a cross-country comparison. Public Choice.

[62] Jurić H, Jansen BES, Gosić N (2011). Croatia: Politics, Legislation, Patients' Rights and Euthanasia. Law, Public Health Care System and Society, Volume 5: Croatia: Politics, Legislation, Patient’s Rights and Euthanasia.

[63] Serrão D, Council of European Publishing (2004). Portugal–Euthanasia: not ethically permissible. Ethical Eye – Euthanasia Vol II – National and European perspectives, Volume 2.

[64] Balint K, Benaya EK, Hansen MK, Nielsen MR, Ørnstrup CA (2014). Euthanasia in Danish print media.

[65] Hildén HM, Honkasalo ML (2006). Unethical bunglers or humane professionals? Discussions in the media of end-of-life treatment decisions. Commun Med.

[66] McInerney F (2006). Heroic frames: discursive constructions around the requested death movement in Australia in the late-1990s. Soc Sci Med.

[67] Atwood-Gailey E (2003). Write to death : news framing of the right to die conflict, from Quinlan's coma to Kevorkian's conviction.

[68] Schwartz KD, Lutfiyya ZM (2009). ‘What lay ahead…’: a media portrayal of disability and assisted suicide. JORSEN.

[69] Birenbaum-Carmeli D, Banerjee A, Taylor S (2006). All in the family: media presentations of family assisted suicide in Britain. Soc Sci Med.

[70] Hausmann E (2004). How press discourse justifies euthanasia. Mortality.

[71] Van Brussel L, Van Landeghem P, Cohen J (2014). Media coverage of medical decision making at the end of life: a Belgian case study. Death Studies.

[72] Goold SD, Klipp G (2002). Managed care members talk about trust. Soc Sci Med.

[73] Rotter JB (1971). Generalized expectancies for interpersonal trust. Am Psychol.

[74] Somerville M (2014). Death Talk: The Case Against Euthanasia and Physician-Assisted Suicide. 2nd editon.

[75] Sudore RL, Landefeld CS, Pantilat SZ, Noyes KM, Schillinger D (2008). Reach and impact of a mass media event among vulnerable patients: the Terri Schiavo story. J Gen Intern Med.

[76] Green-Pedersen C (2007). The conflict of conflicts in comparative perspective - Euthanasia as a political issue in Denmark, Belgium, and the Netherlands. Comp Polit.

[77] Pollock JC, Yulis SG (2004). Nationwide newspaper coverage of physician-assisted suicide: a community structure approach. J Health Commun.

[78] Marques MD, Critchley CR, Walshe J (2014). Attitudes to genetically modified food over time: How trust in organizations and the media cycle predict support. Public Underst Sci.

[79] Hossain F, Onyango B (2004). Product attributes and consumer acceptance of nutritionally enhanced genetically modified foods. Intl J Consum Stud.

[80] Priest SH, Bonfadelli H, Rusanen M (2003). The "trust gap" hypothesis: predicting support for biotechnology across national cultures as a function of trust in actors. Risk Anal.

[81] Gutteling J, Hanssen L, van der Veer N, Seydel E (2006). Trust in governance and the acceptance of genetically modified food in the Netherlands. Public Underst of Sci.

[82] Kim D, Baum CF, Ganz ML, Subramanian SV, Kawachi I (2011). The contextual effects of social capital on health: a cross-national instrumental variable analysis. Soc Sci Med.

[83] Poortinga W (2006). Social capital: an individual or collective resource for health?. Soc Sci Med.

[84] Inglehart R (1997). Modernization and postmodernization: Cultural, economic, and political change in 43 societies.

[85] Davis DW (2000). Individual level examination of postmaterialism in the US: political tolerance, racial attitudes, environmentalism,-aand participatory norms. Polit Res Quart.

[86] Inglehart R, Welzel C (2005). Modernization, cultural change, and democracy: The human development sequence.

[87] Hox JJ (2010). Multilevel analysis: Techniques and applications (2nd edition).

[88] Cohen J, Van Wesemael Y, Smets T, Bilsen J, Deliens L (2012). Cultural differences affecting euthanasia practice in Belgium: one law but different attitudes and practices in Flanders and Wallonia. Soc Sci Med.

[89] Miccinesi G, Fischer S, Paci E, Onwuteaka-Philipsen BD, Cartwright C, van der Heide A, Nilstun T, Norup M, Mortier F, EURELD consortium (2005). Physicians' attitudes towards end-of-life decisions: a comparison between seven countries. Soc Sci Med.

[90] van Delden JJ, Battin MP (2008). Euthanasia: not just for rich countries.

[91] Gordijn B, Janssens R (2000). The prevention of euthanasia through palliative care: new developments in The Netherlands. Patient Educ Couns.

[92] Putnam RD (2001). Bowling Alone.

[93] Uslaner EM (2002). The moral foundations of trust.

[94] Kawachi I, Kennedy BP, Wilkinson RG (1999). Crime: social disorganization and relative deprivation. Soc Sci Med.

[95] Roh S, Lee J-L (2013). Social capital and crime: a cross-national multilevel study. Int J Law Crime Justice.

[96] Sampson RJ, Raudenbush SW, Earls F (1997). Neighborhoods and violent crime: a multilevel study of collective efficacy. Science.

[97] Watkins P (2005). Euthanasia–the erosion of trust?. Clin Med.

[98] Hall M, Trachtenberg F, Dugan E (2005). The impact on patient trust of legalising physician aid in dying. J Med Ethics.

[99] Lindblad A, Löfmark R, Lynöe N (2009). Would physician-assisted suicide jeopardize trust in the medical services? An empirical study of attitudes among the general public in Sweden. Scand J Public Health.

[100] Anderson LA, Dedrick RF (1990). Development of the Trust in Physician scale: a measure to assess interpersonal trust in patient-physician relationships. Psychol Rep.

[101] Rogers JR (1996). Assessing right to die attitudes: a conceptually guided measurement model. J Soc Issues.

[102] Wasserman J, Clair JM, Ritchey FJ (2005). A scale to assess attitudes toward euthanasia. Omega-J Death Dying.

[103] Stronegger WJ, Burkert NT, Grossschadl F, Freidl W (2013). Factors associated with the rejection of active euthanasia: a survey among the general public in Austria. BMC Med Ethics.

[104] Ives J (2014). A method of reflexive balancing in a pragmatic, interdisciplinary and reflexive bioethics. Bioethics.

[105] Salloch S, Schildmann J, Vollmann J (2012). Empirical research in medical ethics: how conceptual accounts on normative-empirical collaboration may improve research practice. BMC Med Ethics.

[106] Fetchenhauer D, Dunning D (2009). Do people trust too much or too little?. J Econ Psychol.

[107] Fetchenhauer D, Dunning D (2010). Why so cynical?: asymmetric feedback underlies misguided skepticism regarding the trustworthiness of others. Psychol Sci.

[108] Ackerman F, Battin M (1998). Assisted suicide, terminal illness, severe disability, and the double standard. Physician Assisted Suicide: Expanding the Debate.

[109] Huxtable R, Möller M (2007). Setting a principled boundary’? Euthanasia as a response to ‘life fatigue. Bioethics.

[110] Huxtable R: Logical separation? Conjoined twins, slippery slopes and resource allocation. J Soc Welf Fam L. 23: 459-471.

[111] Gaskell G, Allum N, Bauer M, Durant J, Allansdottir A, Bonfadelli H, Boy D, de Cheveigne S, Fjaestad B, Gutteling JM, Hampel J, Jelsøe E, Correia Jesuino J, Kohring M, Kronberger N, Midden C, Hviid Nielsen T, Przestalski A, Rusanen T, Sakellaris G, Torgersen H, Twardowski T, Wagner W (2000). Biotechnology and the European public. Nat Biotechnol.

[112] Fenigsen R (1989). A case against Dutch euthanasia. Hatings Cent Rep.

[113] Rietjens JA, Raijmakers NJ, Kouwenhoven PS, Seale C, van Thiel GJ, Trappenburg M, van Delden JJ, van der Heide A (2013). News media coverage of euthanasia: a content analysis of Dutch national newspapers. BMC Med Ethics.

[114] World Health Organisation (2000). The world health report.

[115] O’Brien RM (2007). A caution regarding rules of thumb for variance inflation factors. Qual Quant.

[CR116] The pre-publication history for this paper can be accessed here:http://www.biomedcentral.com/1472-6939/15/86/prepub

