# Supplementary figures and images for: Trust increases euthanasia acceptance: a multilevel analysis using the European Values Study
Source: BMC Med Ethics. 2014 Dec 20;15:86. doi: 10.1186/1472-6939-15-86 (PMC4289573; doi:10.1186/1472-6939-15-86)

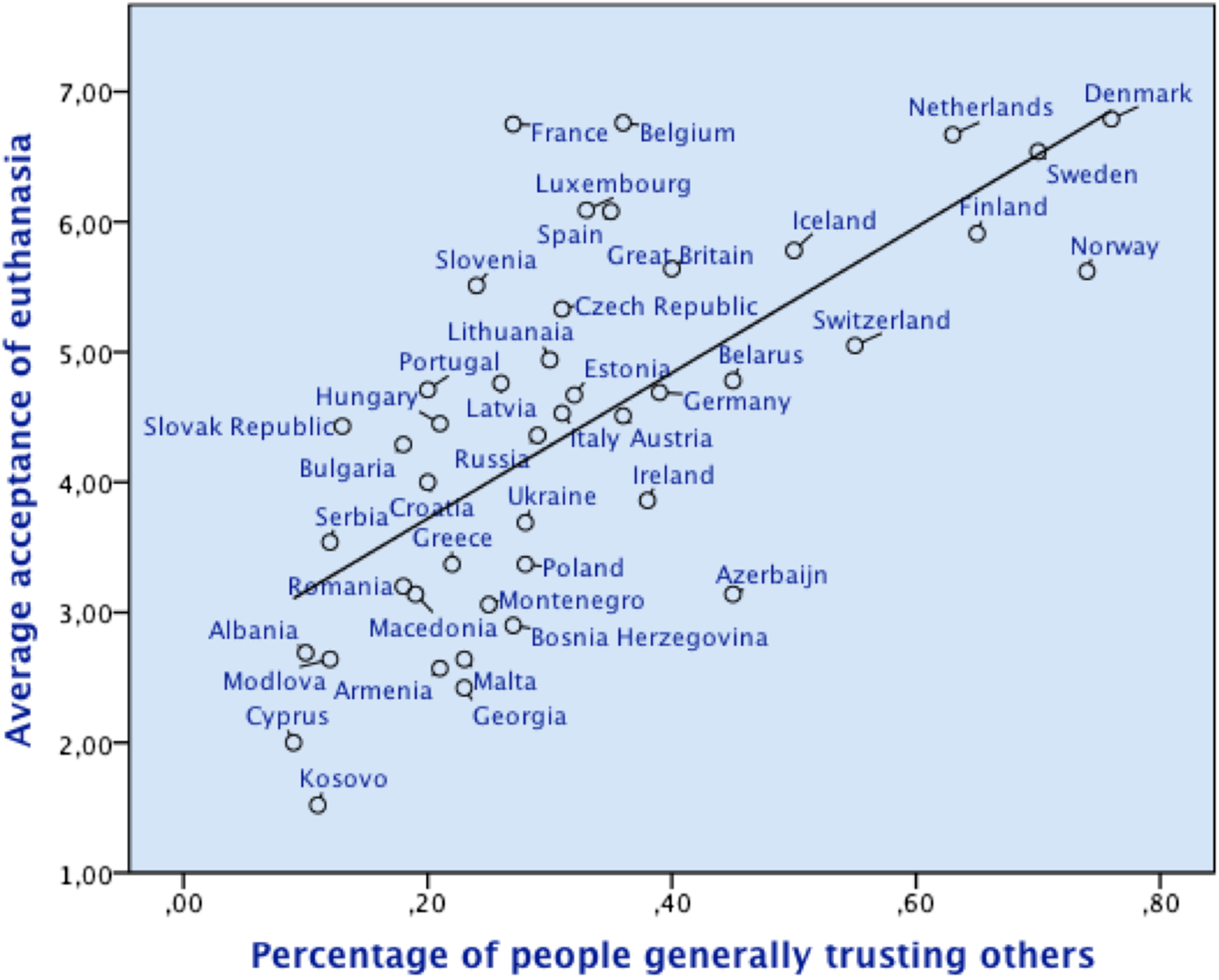

Supplement: Supplementary file 1 — Authors’ original file for figure 1 [file 12910_2014_320_MOESM1_ESM.tiff]
